# Olfactory dysfunction after autoimmune encephalitis depending on the antibody type and limbic MRI pathologies

**DOI:** 10.3389/fneur.2023.1225975

**Published:** 2023-08-25

**Authors:** Martin Hänsel, Henning Schmitz-Peiffer, Antje Hähner, Heinz Reichmann, Hauke Schneider

**Affiliations:** ^1^Department of Neurology, University of Dresden, Dresden, Germany; ^2^Department of Internal Medicine, GZO – Zurich Regional Health Center, Wetzikon, Switzerland; ^3^Smell and Taste Clinic, Department of Otorhinolaryngology, Medical Faculty Carl-Gustav Carus, Technical University of Dresden, Dresden, Germany; ^4^Department of Neurology, Augsburg University Hospital, Augsburg, Germany

**Keywords:** autoimmune encephalitis, olfaction, olfactory dysfunction, threshold discrimination identification test, NMDAR, GAD, LGI1, Caspr2

## Abstract

**Objective:**

Patients’ olfactory function after autoimmune encephalitis (AE) involving limbic structures may be impaired. This study aimed to characterize olfactory function in patients after autoimmune encephalitides.

**Methods:**

A case–control study was performed including 11 AE patients with antibodies against NMDAR (*n* = 4), GAD (*n* = 3), VGKC (*n* = 3) and antibody-negative AE (*n* = 1) and a control group of 12 patients with pneumococcal meningo-encephalitis (PC). In subgroup analyses, AE patients with and without NMDAR-antibodies were compared. Olfactory function was assessed using the Sniffin Sticks test and the resulting TDI-score (threshold, discrimination, identification). Involvement of limbic structures was evaluated on imaging data (MRI). Statistical analyses were performed to test for correlations of TDI-score and MRI results.

**Results:**

The overall olfactory function of the AE-group and the PC-group was comparable (mean TDI 32.0 [CI 27.3–36.7], 32.3 [CI 28.5–36.0)]. The proportions of hyposmic patients were similar compared to the general population. However, AE patients of the non-NMDAR group had significantly lower TDI-scores (28.9 ± 6,8) than NMDAR patients (37.4 ± 3.5) (*p* = 0.046) and a significantly lower discrimination capability than the NMDAR patients (9.9 ± 2.0 vs. 14.5 ± 0.6) (*p* = 0.002). The non-NMDAR patients had significantly more limbic MRI pathologies (6/7) compared to the NMDAR patients (0/4) (*p* = 0.015). Furthermore, a correlation between limbic MRI pathologies and worse capability of smelling discrimination was found (*p* = 0.016, *r* = −0.704, *n* = 11).

**Conclusion:**

Our results indicate that patients with NMDAR autoimmune encephalitis have normal long term olfactory function. However, patients with non-NMDAR autoimmune encephalitis appear to have a persistently impaired olfactory function, probably mediated by encephalitic damage to limbic structures.

## Introduction

Since the first description of the N-methyl-D-aspartate receptor encephalitis (NMDAR) in 2007 (1), autoimmune encephalitides (AE) have been increasingly recognized as important clinical entities in the spectrum of immune mediated neurological diseases. In patients with AE, clinical syndromes vary and are often associated with specific anti-neuronal antibodies. Limbic encephalitis is the predominant syndrome and involves the medial temporal lobe with its hippocampal structures, leading to neurocognitive impairment and psychiatric symptoms (2, 3). However, less is known about the potential involvement of other limbic functions in AE patients, particularly the olfactory system. AE may affect the piriform cortex in the mesial temporal lobe (primary olfactory cortex), and the orbitofrontal cortex (secondary olfactory cortex). In 2019 Geran et al. showed in a cohort of 32 AE patients, that olfactory dysfunction is present in 75% of AE patients (4). In another work by Morano et al., olfactory dysfunction was found in 15 of 19 AE patients (5). The heterogeneity of the groups was high with 12 different antibody-types including seronegative AE in the first sample (4) and 5 different antibody-types in the second sample (5). Considering that different AE antibody-types are associated with a spectrum of clinical syndromes, olfactory function may be differentially affected in known AE subtypes. Furthermore, it is unclear whether limbic MRI pathologies in AE are associated with impaired olfaction.

The objective of the study was to characterize the olfactory function after recovery from AE and find correlating MRI patterns in the acute phase of patients with AE and to compare subgroups of patients with NMDAR antibodies and patients with non-NMDAR antibodies.

## Methods

### Study participants

Patients with autoimmune encephalitis were enrolled in this single-centre case–control study and compared with sex– and age-matched patients with pneumococcal meningo-encephalitis (PC), treated at the University Hospital Dresden, Germany between 1st January 2002 and 31th March 2016. The process of patient inclusion is described in detail in the supplement (Supplementary Figure S1). The diagnosis of the AE was based on the clinical course and positive antibody-findings in serum and / or cerebrospinal fluid, and in line with the current diagnostic criteria for definite AE (6). We recruited patients with N-methyl-D-aspartate receptor (NMDAR) antibodies, glutamic acid decarboxylase (GAD) antibodies, voltage-gated potassium channels (VGKC) antibodies and an antibody-negative patient. For subgroup analyses, patients were divided in a group testing positive for NMDAR antibodies (NMDAR) and a group of AE patients with the other antibody results (non-NMDAR: VGKC, GAD, antibody-negative). For AE patients diagnosed in 2010 and 2011, antibodies to NR1/NR2B heteromers of the NMDAR were detected by indirect immunofluorescence on NR1/NR2B transfected human embryonic kidney cells (7, 8). In all other AE patients, antibodies were detected by indirect immunofluorescence on commercially available mouse brain tissue and cell-based assays (Euroimmun, Lübeck, Germany) (9) (Supplementary). After written informed consent, clinical and imaging data of study participants were retrieved and analysed. The study-visits were made in person between 1st August 2016 and 31th December 2016 and consisted in clinical examination as well as testing of olfactory and cognitive functions.

### Inclusion criteria

Patients had to be aged 18 or older, non-pregnant and give consent to study participation. Further inclusion criteria of the control group of PC patients were treatment at a critical care unit, surgical treatment of infectious focus (sinugen, otogen), age at diagnosis 40 ± 15 years, and the detection of streptococcus pneumoniae in blood cultures.

### Olfactory function testing

The olfactory function was tested by “Sniffin’Sticks” (Heinrich Burghart GmbH, Wedel, Germany) (10) in three steps: threshold (T), discrimination (D) and identification (I) (TDI test). The odour threshold was measured using a 16-step three-alternate forced-choice staircase paradigm. The patients had to identify the pen with n-butanol in increasing concentration out of the other two pens filled without odorant. Concentration was increased if one of the blanks was chosen and decreased if the correct pen was identified twice in a row. The mean of the last 4 of a total of 7 reversal points was used as detection threshold (ranging from 1 to 16). For discrimination, patients had to identify the one pen with different odour out of three pens in 16 transits. When testing for odour identification, 16 pens containing common odours were offered. The patient had to identify each of the odorants from a list of four descriptors. The maximum TDI-score is 48 points, a score > 28 indicates normosmia (in patients >55 years), >16–28 points hyposmia and ≤ 16 indicates anosmia (10) (Supplementary Table S1).

### Further investigations

To identify influencing confounders of the olfactory system, every patient answered a questionnaire before olfactory testing (Supplementary Table S2). The patients were asked about possible chronic sinonasal problems (e.g., allergies, polyps, operations at the sinonasal tracts), medical treatment (e.g., calcium-antagonists, antibiotics, corticosteroids, nose drops) or chronic diseases (e.g., Parkinsonism, Alzheimer disease, Multiple sclerosis). Every patient was tested with the Hospital anxiety and depression scale (HADS) to screen for major depression (D: cut off >10 points) and anxiety (A: cut off >10 points) at the time of testing (11). Furthermore, the Montreal Cognitive Assessment (MoCA) was used to screen for mild cognitive impairment (cut off: <26 points) as a potentially influencing factor when testing olfactory dysfunction (12).

Imaging data (brain MRI), acquired during the initial in-hospital treatment period, were analysed regarding limbic MRI pathologies (e.g., temporomesial, temporobasal, hippocampus and Corpus amygdaloideum) in the FLAIR- and T2 sequences (13). Routine MRI was performed using either Siemens Magnetom Verio (3.0 Tesla), Siemens Magnetom Vision (1.5 Tesla) or GE Signa HDxt (3.0 Tesla). The MRI scanner was selected according to scanner availability in clinical routine. All scans were rated by a senior neuroradiologist.

### Statistical analysis

Metric data were analysed by the unpaired t-test with a confidence-interval of 95% and reported as significant, if *p* < 0.05. Correlation analyses were calculated with the Spearman’s rho and nominal data with Fisher’s exact test. Furthermore, we applied the Mann–Whitney-U-test to analyse significant relations between pathologic MRI-findings and the TDI-score. The effect sizes of the olfactory function were calculated using Cohen’s d. To test the distribution, we used the Shapiro–Wilk test due to the small sample size. The distribution results are included in the Supplementary part. Statistical analysis was done using SPSS (V.23.0, IBM, Armonk, New York, United States).

## Results

### Demographic, clinical, and treatment characteristics in AE and PC patients

11 patients with AE [median age 31 (range 17–74); 8 women] were compared with 12 PC patients [median age 41 (range 26–52); 5 women]. AE-specific antibodies were found in 10 of 11 patients: NMDAR in 4 patients, GAD in 3 patients, and VGKC in 3 patients; in one patient, no AE antibodies were detected (Table 1). 9/11 AE patients got a first-line therapy with methylprednisolone: 4/9 patients in combination with intravenous immunoglobulins, 2/9 patients in combination with immunoadsorption. 4/9 patients with first line therapy required a second-line therapy with Rituximab. 2/11 patients were found to have GAD antibodies several years later after symptom onset and were therefore initially treated with anti-epileptic drugs only. The mean duration of hospital stay in the AE group was 27.1 (±15.8) days and 15.7 (±4.1) days in the PC control group (*p* = 0.039). The median time between diagnosis and follow up -visit was 33 (IQR 20.0–85.0) months in the AE group and 96 (IQR 64.0–113.8) months at the control-group (*p* = 0.008). A tumour (incidentaloma) was found after diagnosis in 3 of 11 patients, once in a NMDAR patient and twice in VGKC patients. In one patient with GAD, focal cortical dysplasia type Ia and mild cortical dysplasia type II was diagnosed in the right hippocampus 4 years before GAD antibodies were detected.

**Table 1:** Patient characteristics in AE and PC groups.

	AE group	PC group	Significance
Patients, *n*	11	12	–
Ratio female: male	8: 3	5: 7	*p* = 0.214
Median age at diagnosis (yrs), median, [range]	31 [17–74]	41 [26–52]	*p* = 0.984
Antibodies against			
synaptic receptors: NMDAR	4	–	–
ion channels: VGKC (LGI1, Caspr2)	3	–	–
intracellular antigens: GAD	3	–	–
Antibody negative	1	–	–
Patients with comorbidities at diagnosis	11	11	*p* = 1.000
mRS, median [range]			
Acute	3 [2–5]	5 [2–5]	*p* = 0.014
Follow up	1 [0–1]	1 [0–4]	*p* = 0.236
Acute therapy			
IV corticosteroids	9	–	–
Intravenous immunoglobulin	4	–	–
Immunadsorption	2	–	–
Second line therapy			
Rituximab	4	–	–
Hospitalisation time (d), mean, [range]	27.1 [6–48]	15.7 [10–25]	*p* = 0.039
Time from diagnosis to follow up (mo), median, [range]	33 [11–95]	96 [26–132]	*p* = 0.008
Limbic MRI pathologies, n	6	0	*p* = 0.018
HADS-D, median [range]	3 [1–12]	5 [1–17]	*p* = 0.229
MoCA, median [range]	27.0 [17–29]	27.5 [22–30]	*p* = 0.281

AE, autoimmune encephalitis; NMDAR, N-methyl-D-aspartate Receptor; Caspr2, contactin-associated protein-like 2; GAD, glutamate acid decarboxylase; HADS-D, Hospital anxiety and depression scale; LGI1, leucine-rich glioma inactivated 1; MoCA, Montreal Cognitive Assessment; mRS, modified Ranking Scale; PC, pneumococcal meningo-encephalitis; VGKC, voltage-gated potassium channels.

### Olfactory function in AE and PC patients

Olfactory testing revealed that 3/11 patients with AE and 2/12 patients with PC were hyposmic (*p* = 0.915). The mean TDI-score in the AE and PC groups were 32.0 (±7.1; CI 27.3–36.7) and 32.3 (±5.9; CI 28.5–36.0) respectively. There were no significant differences in threshold, discrimination, identification and TDI (*p* = 0.915) between both groups (Table 2). The questionnaires on olfactory functions showed no significant differences between both groups, and abnormalities were on the level of the standard population (14, 15).

**Table 2:** Olfactory function in AE and PC groups.

	AE group	PC group	Significance	Effect size
Normosmia, n (%)	8 (72.7)	10 (83.3)	*p* = 0.236	–
Hyposmia, n (%)	3 (27.3)	2 (16.7)	*p* = 0.236	–
TDI score, mean (SD)	32.0 (7.1)	32.3 (5.9)	*p* = 0.915	d = 0.496
Threshold, mean (SD)	7.7 (3.1)	7.7 (3.6)	*p* = 0.989	d = 0.001
Discrimination, mean (SD)	11.6 (2.8)	12.0 (1.3)	*p* = 0.621	d = 0.519
Identification, mean (SD)	12.7 (2.6)	12.6 (3.0)	*p* = 0.905	d = 0.005

AE, autoimmune encephalitis; PC, pneumococcal meningo-encephalitis; SD, standard deviation; TDI, threshold discrimination identification test.

### MRI findings in AE and PC patients

**Figure 1:**
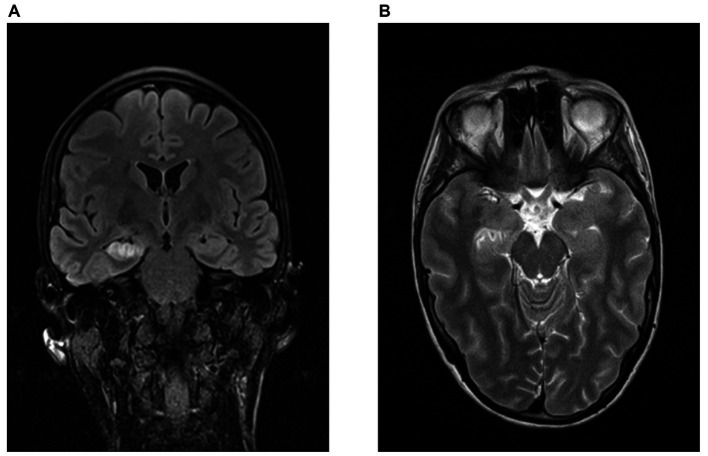
Limbic MRI pathologies in a patient with GAD-antibodies. Representative MRI-findings of a patient with right hippocampal necrosis and space-occupying effects temporomesial at the amygdala complex, hippocampus and nucleus accumbens on the right. **(A)** FLAIR-sequence, coronary. **(B)** T2-weighted sequence, transversal (source: University Hospital Dresden, Department of Neuroradiology).

In the AE group, 6/11 patients had limbic MRI pathologies at the time of initial hospital treatment, which persisted in all follow-up MRIs in these patients [median time of 4 (range 1–71) months] (Figure 1). In contrast, the remaining 5 AE patients and 7/7 PC patients had no limbic MRI pathologies both in the early phase and during follow-ups (*p* = 0.018). Patients with limbic MRI pathologies had significantly lower olfactory discrimination capabilities (*p* = 0.008, *r* = −0.590, *n* = 19). The group with limbic pathologies had a mean discrimination score of 9.8 (±2.2), the group without a score of 12.8 (±1.7).

### Further investigations in AE and PC patients

There were no significant differences in the median HADS-D score (*p* = 0.229) between the both groups [AE = 3 (range 1–12); PC = 5 (range 1–17)]. One GAD patient within the AE group and 2 patients of the PC group had an increased HADS-D score (> 10 points). The median HADS-A score (*p* = 0.775) in the AE group was 5 [range 1–13] and in the PC group 5.5 [range 2–12]. In 10 AE patients and 10 PC patients, no significantly elevated HADS-A scores were identified. The median MoCA-score was comparable in AE patients and PC patients [27 (range 17–29) vs. 27.5 (range 22–30), *p* = 0.281]. 8/11 AE patients had a normal MoCA score (≥ 26 points) and 8/12 PC patients reached a normal MoCA score, without significant differences between both groups (*p* = 1.000).

### Demographic and treatment characteristics in NMDAR and non-NMDAR patients

At disease onset, patients of the NMDAR group (4/4 women, median age 19 years, range 17–29) were significantly younger compared to patients of the non-NMDAR group (4/7 women, median age 58 years, range 23–74) (*p* = 0.006). The median duration of hospital stay differed significantly in-between both groups (NMDAR vs. non-NMDAR: 42 days vs. 15 days) (*p* = 0.023) (Table 3).

**Table 3:** Patient characteristics in NMDAR and non-NMDAR groups.

	NMDAR patients	non-NMDAR patients	Significance
Patients, n	4	7	–
Ratio female: male	4: 0	4: 3	*p* = 0.236
Median age at diagnosis (yrs), median, [range]	19 [17–29]	58 [23–74]	*p* = 0.006
Patients with comorbidities at diagnosis, *n*	4	7	*p* = 1.000
mRS, median [range]			
Acute	3 [3–5]	2 [2–4]	*p* = 0.166
Follow up	0 [0–1]	1 [0–1]	*p* = 0.166
First line immunotherapy			
IV corticosteroids	4	5	*p* = 0.491
Intravenous immunoglobulin	3	1	*p* = 0.088
Immunadsorption	0	2	*p* = 0.491
Second line immunotherapy			
Rituximab	3	1	*p* = 0.088
Hospitalisation time (d), mean, [range]	40.5 [33–45]	19.4 [6–48]	*p* = 0.023
Time from diagnosis to follow up (mo), median, [range]	83 [20–95]	28 [11–85]	*p* = 0.121
Limbic MRI pathologies, n	0	6	*p* = 0.015
HADS-D, median [range]	2.5 [1–4]	3.0 [1–12]	*p* = 0.367
MoCA, median [range]	27.5 [26–28]	26.0 [17–29]	*p* = 0.186

NMDAR, N-methyl-D-aspartate Receptor; HADS-D, Hospital anxiety and depression scale; MoCA, Montreal Cognitive Assessment; mRS, modified Ranking Scale; non-NMDAR, non-N-methyl-D-aspartate Receptor.

### Olfactory function in NMDAR and non-NMDAR patients

**Figure 2:**
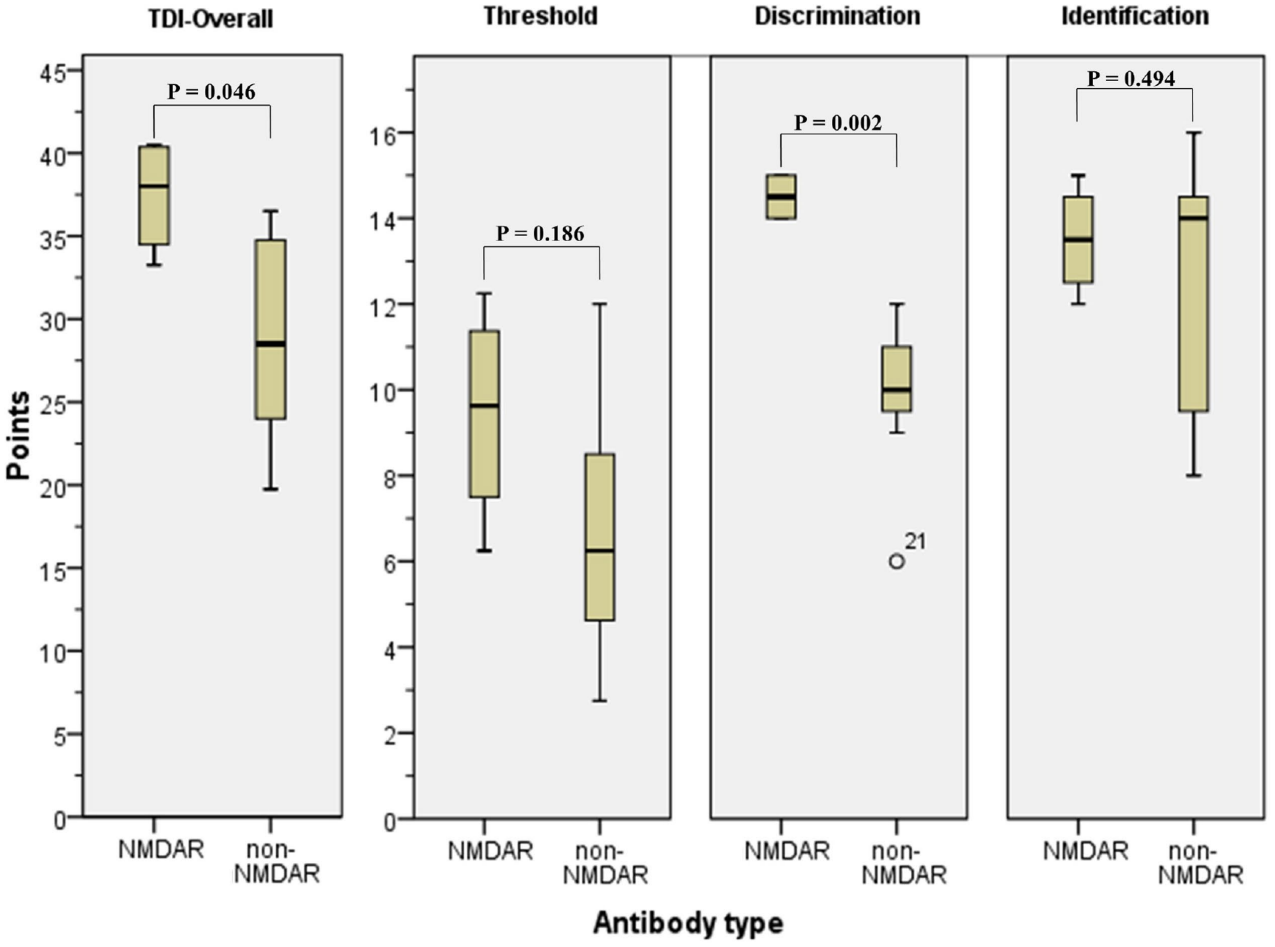
Threshold Discrimination Identification Test in NMDAR and non-NMDAR patients. TDI-test in NMDAR patients and non-NMDAR patients. NMDAR, N-methyl-D-aspartate Receptor; non-NMDAR, non-N-methyl-D-aspartate Receptor; TDI, threshold discrimination identification test.

All patients of the NMDAR group were normosmic, whereas 3/7 patients of the non-NMDAR group were hyposmic (*p* = 0.236). The mean TDI-score of the NMDAR group was significantly higher than at the non-NMDAR group (37.4 ± 3.5 vs. 28.9 ± 6.8) (*p* = 0.046) (Figure 2). There were no significant differences between the NMDAR and non-NMDAR group in threshold (9.4 ± 2.6 vs. 6.8 ± 3.2; *p* = 0.182) and identification (13.5 ± 1.3 vs. 12.3 ± 3.2; *p* = 0.400). The discrimination was significantly higher in the NMDAR group (14.5 ± 0.6) in comparison to the non-NMDAR group (9.9 ± 2.0) (*p* = 0.002) (Table 4).

**Table 4:** Olfactory function in NMDAR and non-NMDAR groups.

	NMDAR patients	non-NMDAR patients	Significance	Effect size
Normosmia, *n* (%)	4 (100)	4 (57.1)	*p* = 0.640	-
Hyposmia, *n* (%)	0 (0)	3 (42.9)	*p* = 0.640	-
TDI score, mean (SD)	37.4 (3.5)	28.9 (6.8)	*p* = 0.046	d = 1.139
Threshold, mean (SD)	9.4 (2.6)	6.8 (3.2)	*p* = 0.186	d = 0.324
Discrimination, mean (SD)	14.5 (0.6)	9.9 (2.0)	*p* = 0.002	d = 0.562
Identification, mean (SD)	13.5 (1.3)	12.3 (3.2)	*p* = 0.494	d = 1.198

NMDAR, N-methyl-D-aspartate Receptor; non-NMDAR, non-N-methyl-D-aspartate Receptor; SD, standard deviation; TDI, threshold discrimination identification test.

### MRI findings in NMDAR and non-NMDAR patients

In NMDAR patients, no limbic MRI pathologies were present at disease onset and during follow up. In comparison, 6/7 patients of the non-NMDAR group had limbic MRI pathologies at onset and during follow up [mean follow up 13.3 (±21.7) months] (*p* = 0.015). All 3 hyposmic patients had limbic MRI pathologies. In patients with autoimmune encephalitis there was a significant correlation between a worse discrimination capability and limbic MRI pathologies (*p* = 0.016, *r* = −0.704, *n* = 11).

## Discussion

Several cerebral structures, primarily the limbic system, are functionally affected in antibody-mediated autoimmune encephalitis. Olfactory dysfunction may be associated with AE. This has recently been suggested by two studies investigating olfactory function in AE patients (4, 5). In contrast to these studies, which reported impaired olfactory function in most AE patients, in our AE cohort, overall olfactory function was not significantly impaired compared to a standard population. However, in our study, we observed significant differences in olfactory function depending on the AE antibody-type and an association with acute phase MRI lesions in the limbic system was demonstrated. Normal olfaction in NMDAR patients during long term follow-up contrasted with impaired olfactory function in patients with other AE antibodies, especially anti-GAD which demonstrated worse discrimination capability and overall-olfaction. Moreover, we found a significant association between MRI lesions in the limbic system and olfactory dysfunction in AE, also predominantly in non-NMDAR patients.

The different antibodies causing AE in our cohort are directed against surface or intracellular targets, which might explain the different results for NMDAR and non-NMDAR patients in our study. Antibodies against the NMDAR cause a selective and reversible decrease of synaptic NMDAR by antibody-mediated capping and internalization of surface NMDARs (16). There is usually no irreversible structural damage of neurons, which explains the therapy-response in NMDAR patients with early immunosuppressive treatment (17). In contrast, in patients with GAD antibodies, targeting intracellular antigens, and VGKC antibodies targeting ion channels, immunotherapies are less effective (18, 19). These mechanisms might also explain the observed normal olfactory function of NMDAR patients and olfactory deficits in AE patients with non-NMDAR antibodies. However, larger cohorts of AE patients with defined subgroups should be evaluated in future studies to further clarify the impact of different antibody-types on olfaction.

In contrast to our results, Geran et al. reported olfactory dysfunction in 24 of 32 AE patients but did not observe an association with different antibody-types (4). Although there are methodological similarities between both studies, an important difference is the time of olfaction measurement. The median follow-up in our study was 33 ± 34 months after diagnosis, whereas the testing at Gerans group was 18 ± 13 months after diagnosis. We postulate that overall clinical recovery may include olfactory function recovery over time, especially in patient with NMDAR and other cell surface-active antibodies. In future studies, sequential measurements of clinical and olfactory functions after AE should be performed.

Morano et al. detected olfactory dysfunction in 15/19 of AE patients (5). However, because of the different test-design used (Brief Smell Identification Test, B-SIT), only the olfactory identification can be compared. In contrast, no AE patient from our cohort presented an impairment in olfactory identification. The median follow-up time was similar in both studies (33 vs. 37 month), however more than half of patients from Morano et al. were seronegative (10/19), greatly limiting possible comparison of both cohorts.

The observed high rate of limbic MRI pathologies in patients with non-NMDAR encephalitis, which also present a higher rate of olfactory dysfunction, suggests a causative and lasting damage of olfaction-related structures within or associated with the limbic system. In the non-NMDAR group, affected structures in the limbic system included the Corpus amygdaloideum, hippocampus and the temporomesial parenchyma. These MRI pathologies were observed in all patients of the non-NMDAR group and none of the NMDAR group. To our knowledge, other studies reporting MRI data and olfaction data in AE patients are not available. In future studies MRI-based, volumetric measurements of the olfactory bulb should be considered, as the size and volume of the olfactory bulb is a morphological indicator of the olfactory function (20, 21) and correlates with the olfactory function (22). Furthermore, sequential MRI measurements, together with sequential clinical and olfactory testing, might provide further insights in olfactory function after autoimmune encephalitis.

This study has several limitations: First, the small size of our cohort, and second, the heterogeneity of AE antibody-types. Because of the generally low incidence of AE (23), further studies on olfaction in AE should be planned as multi-centre, prospective case–control-studies in larger cohorts. Subgroups should include sufficiently large numbers of patients with antibodies to surface and non-surface targets. Third, we only evaluated MR imaging of the early treatment phase. Future studies should include sequential MRI-based examinations of limbic structures and measurements of the olfactory bulb. Fourth, an otorhinolaryngoscopic examination with a nasal endoscope was not made and should be included in future studies. Fifth, we cannot exclude a possible influence of mild cognitive impairment, severe depression and former temporomesial resection in 2 hyposmic GAD patients, which may lead to other conclusions for non-NMDAR patients of our cohort (11, 12, 24, 25). Sixth, the mean age of the non-NMDAR patients was significantly higher. An impact of the higher age with worse olfactory function and regeneration is possible; age correction was not used because of the small sample size. Seventh, given the small sample sizes, we did not perform sensitivity analyses. Eighth, we included one antibody-negative AE patient, fulfilling diagnostic AE criteria, into the non-NMDAR group, as we preferentially aimed to characterize NMDAR and non-NMDAR subgroups. However, we cannot exclude that AE in seronegative patients is mediated by NMDAR-like or other yet unknown antibodies, and that this may lead to different effects on olfactory function compared to non-NMDAR patients. Furthermore, although meanwhile established, a distinction between anti-LGI1 patients and anti-Caspr2 patients in VGKC-positive patients of our cohort was not made at the time of diagnosis and later on (26).

Certainly, Herpes-simplex-virus (HSV) encephalitis patients which present very similar clinical presentation, pathophysiological aspects and known olfactory impairment in a proportion of patients would be an elegant control population (27–29). Considering the very low incidence of HSV encephalitis and the known higher mortality compared to AE, we feared not being able to recruit enough control subjects. As an alternative control-group, we chose PC patients which are known to present similar levels of olfactory dysfunction after recovery as the normal population (30). Patients with HSV encephalitis could serve as control-group in future larger prospective studies.

## Conclusion

In our small cohort study on olfaction in patients after autoimmune encephalitis we found a persistent olfactory dysfunction in a subgroup of AE patients with antibodies to intracellular antigens and membrane channels, but not in patients with NMDAR antibodies directed against a neuronal cell surface target. Furthermore, early limbic MRI pathologies were detected in all non-NMDAR patients, but not in NMDAR patients, suggesting a lasting, causative structural defect of parts of the limbic system involved in olfaction in non-NMDAR patients. Future studies on olfaction in AE patients should include larger cohorts with predefined antibody-subgroups and include sequential MRI and olfaction measurements.

## Data availability statement

The raw data supporting the conclusions of this article will be made available by the authors, without undue reservation.

## Ethics statement

The ethical approvement was given by the Ethics Committee of the University of Dresden [EK 104032016, EK 125042012, and EK 122032011]. The studies were conducted in accordance with the local legislation and institutional requirements. The participants provided their written informed consent to participate in this study.

## Author contributions

MH: conception and design of the study, acquisition and analysis of data, interpretation of data, and drafting significant portion of manuscript and figures. HS-P and HR: conception and design of the study and critical revision of manuscript for intellectual content. AH: conception and design of the study, analysis of data and critical revision of manuscript for intellectual content. HS: conception and design of the study, analysis of data, drafting significant portion of manuscript and figures, study supervision, and critical revision of manuscript for intellectual content. All authors contributed to the article and approved the submitted version.

## Funding

The Article Processing Charges (APC) were funded by the joint publication funds of the TU Dresden, including Carl Gustav Carus Faculty of Medicine, and the SLUB Dresden as well as the Open Access Publication Funding of the DFG.

## Conflict of interest

The authors declare that the research was conducted in the absence of any commercial or financial relationships that could be construed as a potential conflict of interest.

## Publisher’s note

All claims expressed in this article are solely those of the authors and do not necessarily represent those of their affiliated organizations, or those of the publisher, the editors and the reviewers. Any product that may be evaluated in this article, or claim that may be made by its manufacturer, is not guaranteed or endorsed by the publisher.
