# Supplementary material for: Olfactory dysfunction after autoimmune encephalitis depending on the antibody type and limbic MRI pathologies
Source: Front Neurol. 2023 Aug 25;14:1225975. doi: 10.3389/fneur.2023.1225975 (PMC10486887; doi:10.3389/fneur.2023.1225975)
Supplement: Supplementary file 1 [file Data_Sheet_1.docx]

Supplementary Material

Olfactory dysfunction after autoimmune encephalitis depending on the antibody type and limbic MRI pathologies

Martin Hänsel^1,2^, Henning Schmitz-Peiffer^1^, Antje Hähner^3^, Heinz Reichmann^1^, Hauke Schneider ^1,4*^

*** Correspondence:** Corresponding Author: [hauke.schneider@uk-augsburg.de](mailto:hauke.schneider@uk-augsburg.de)

**1. Methods**

**Detection of antibodies**

For AE patients diagnosed in 2010 and 2011, antibodies to NR1/NR2B heteromers of the NMDAR were detected by indirect immunofluorescence on NR1/NR2B transfected human embryonic kidney cells (7, 8). In all other AE patients, antibodies were detected by indirect immunofluorescence on commercially available mouse brain tissue and cell-based assays (Euroimmun, Lübeck, Germany) (9). The assays consisted of transfected human embryonic kidney (HEK 293) with plasmids encoding the following neuronal antigens: N-methyl-D-aspartate receptor (NMDAR; consisting of NR1 subunits only), NMDAR (NR1-NR2), glutamic acid decarboxylase (GAD) GAD65, GAD67, leucine-rich glioma inactivated1 protein (LGI1), contactin-associated protein-like2 (CASPR2), AMPAR1, AMPAR2, GABABR, glycine receptor (GlyR).

**Comorbidities**

We combined two categories of comorbidities: autoimmune diseases and other diseases before and during AE and PC. In the autoimmune category, patients had e.g. diabetes mellitus type 1, Morbus Basedow, psoriasis vulgaris, Hashimoto's thyroiditis or atopic dermatitis. Other conditions included adnexitis, hepatic steatosis, bronchial asthma, osteoporosis, syndrome of inappropriate antidiuretic hormone secretion (SIADH), depression, arterial hypertension, prostatic hypertrophy, subdural haematoma and atrioventricular nodal reentrant tachycardia.

**2. Results**

**The distribution of olfactory results**

Threshold, discrimination and TDI-overall were approximately normally distributed in both the AE and PC groups, as assessed by the Shapiro-Wilk test, p > 0.05. Identification was approximately normally distributed for the AE group (p = 0.195) but not for the PC group (p = 0.011). For the NMDAR and non-NMDAR groups, identification and overall TDI were normally distributed as assessed by the Shapiro-Wilk test. Discrimination was normally distributed for the non-NMDAR group (p = 0.182), but not for the NMDAR group (p = 0.024).

# 3. Supplementary Figures and Tables

**Supplementary Table 1**

Algorithm of TDI depending on age

|  | **< 16 years** | **16 - 35 years** | **36 - 55 years** | **> 55 years** |
| --- | --- | --- | --- | --- |
| Normosmia | > 25 | > 32 | > 29 | > 28 |
| Hyposmia | >16-25 | >16-32 | >16-29 | >16-28 |
| Anosmia | ≤ 16 | ≤ 16 | ≤ 16 | ≤ 16 |

Reference: Hummel T, Kobal G, Gudziol H, Mackay-Sim A. Normative data for the "Sniffin' Sticks" including tests of odor identification, odor discrimination, and olfactory thresholds: an upgrade based on a group of more than 3,000 subjects. *Eur Arch Otorhinolaryngol*. (2007) 264:237-43. doi: 10.1007/s00405-006-0173-0

**Supplementary Table 2**

Anamnesis questionnaire

Name, forename: ______________________________ Date of birth: _____

| 1. Do you have chronic issues with your nose? | □ yes - if yes, which one?  rhinorrhea, nasal congestion, sneeze, allergies, polyps, face ache, ……………………………………………………………………….......…  □ no (continue with 7.) |
| --- | --- |
| 2. Since when do you have this problems? | □ less than 3 months □ since 3-24 months  □ more than 2 years  □ always / since I can remember  □ I don´t know |
| 3. How did the problem start? | □ gradually □ suddenly  □ I never could smell anything in my life  □ I don´t know |
| 4. Whereon do you refer this problem to a cause? | □ accident □ cold / infection  □ intake of medication □ operation  □ dry mouth □ dental prothesis  □ nasal breathing / polyps / sinusitis  □ others (please quote) |
| 5. Is your disorder fluctuating or constant? | □ fluctuation  □ constant  □ I don´t know  □ changes under specific conditions – if yes, which one? |
| 6. How much are you impaired by this problem? | □ extrem severe □ severe □ middle  □ moderate □ barely □ not at all |
| 7. How good can you breath in and out with your nose? | □ very good  □ good  □ bad  □ very bad  □ I can not breath through my nose |
| 8. Do you use medication, respectively, did you use following medications? If yes, when? | □ no medication  □ calcium antagonist: □ Nifedipin □ Felodipin  □ Amlodipin □ Diltiazem  □ antibiotics, chemotherapeutics:  □ Amikair □ Terbinafin □ Doxycycklin  □ Methotrexat □ D-Penicillamin  □ chronic use of vasoconstrictive drugs (nosedrops)  □ painkillers: Morphin/Remifentanil  □ anticoagulants: Marcoumar (Falithrom)  □ beta-blocker: e.g. Metoprolol (Beloc Zok)  □ ACE-inhibitors: □ Ramipril, □ Captopril, □ Enalapril  □ others: □ Lovastatin, □ Amitriptylin, □ Amiodaron |
| 9. Do you have chronic diseases? | □ no  □ yes - which one?  □ diabetes mellitus typ II □ arterial hypertension  □ thyroid deficiency □ Addison´s disease  □ Cushing´s disease □ epilepsy  □ schizophrenia □ depression  □ hepatic disease □ kidney disease  □ Sjögren-syndrom □ cancer: …………….. |
| 10. Operations at the head region? | □ no □ yes - which one?  □ paranasal sinuses □ nasal septum  □ nasal polyps □ nasal concha  □ tonsillar □ pharyngeal tonsils  □ middle ear: □ left □ right  □ special dental operations □ others…………….. |
| 11. Flu vaccination? | □ no □ yes - when? |
| 12. Do you smoke? | □ no □ yes - how much? |
| 13. Do you drink alcohol? | □ no □ yes □ sometimes □ regulary |
| 14. Profession? | Are you exposed to dust, gas, chemical (formaldehyde, herbicides, pesticides) in a specific manner? □ no □ yes  If yes, which one?  How many years?  How many hours each day? |
| 16. Family anamnesis | Diseases in you cosanguine family:  - Parkinson´s disease □ no □ yes  - Alzheimer´s disease □ no □ yes  - multiple sclerosis □ no □ yes |

**Supplementary Figure 1**


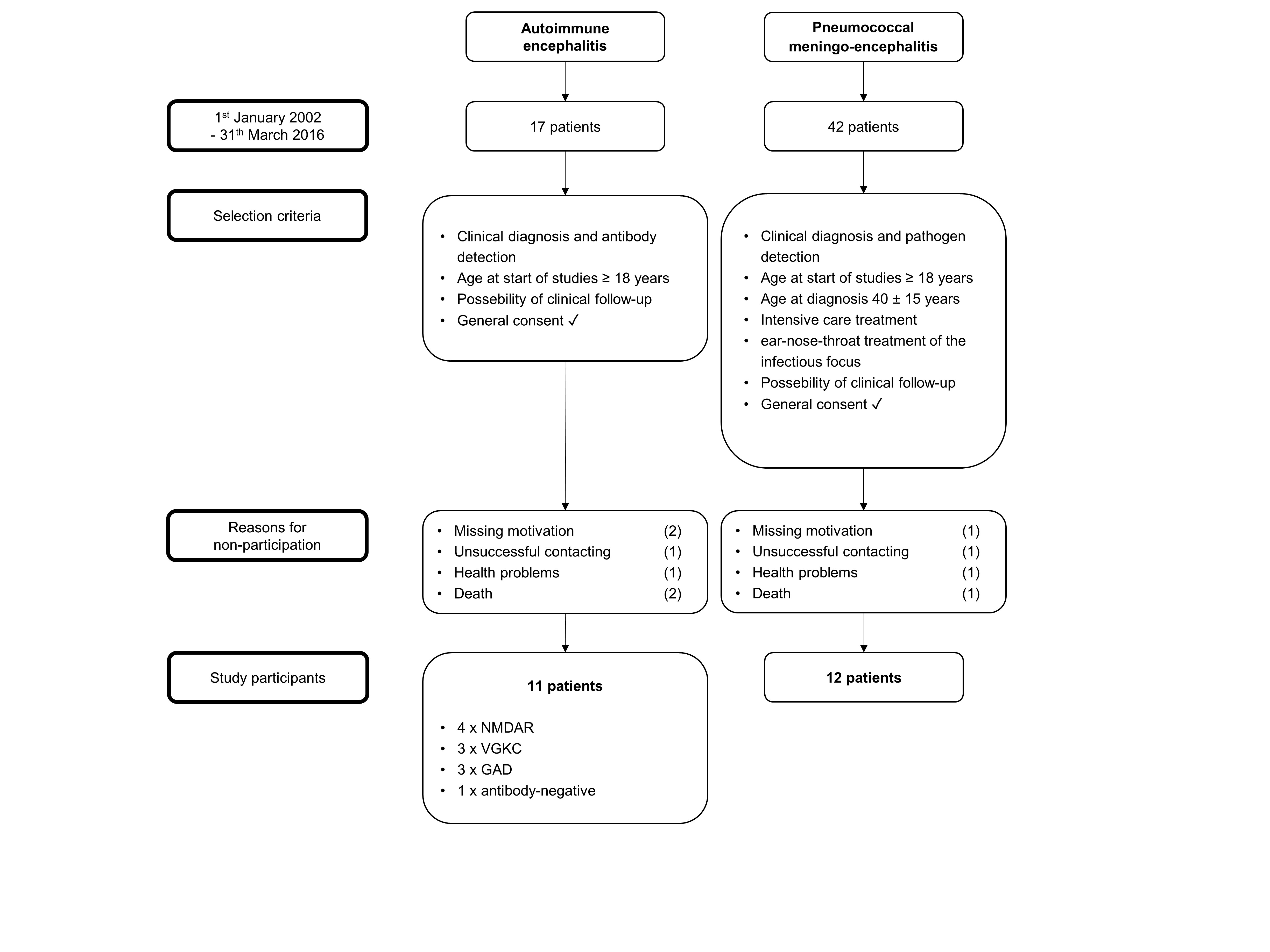
,

**
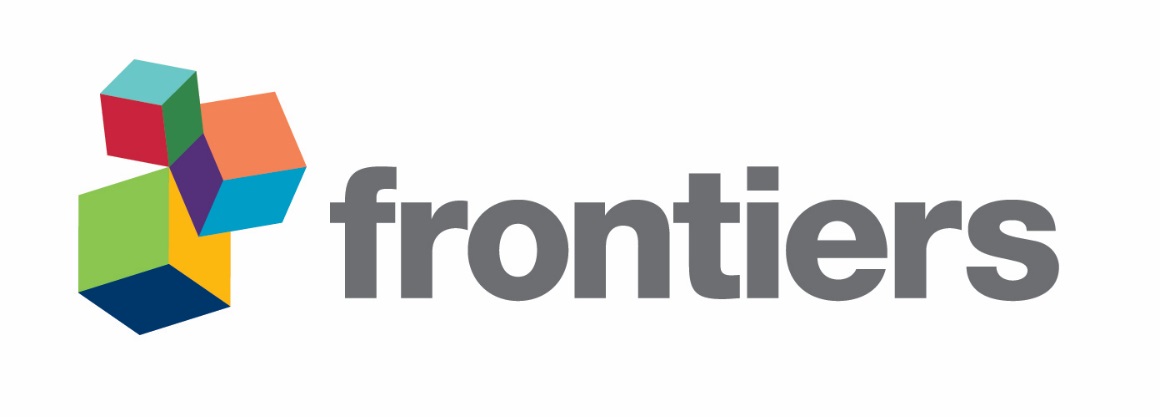
**
